# 4D imaging of fetal right ventricle—feasibility study and a review of the literature

**DOI:** 10.1007/s10554-021-02407-9

**Published:** 2021-09-20

**Authors:** M. Pasieczna, J. Duliban, A. Grzyb, J. Szymkiewicz-Dangel

**Affiliations:** 1 2nd Department Of Obstetrics and Gynecology, Medical University of Warsaw, Karowa Street 2, 00-315 Warsaw, Poland; 2 Department of Cardiology, The Children’s Memorial Health Institute, Warsaw, Poland; 3 Department of Perinatal Cardiology and Congenital Anomalies, The Centre of Postgraduate Medical Education, Warsaw, Poland

**Keywords:** 4D echocardiography, Fetal echocardiography, Right ventricle, Fetal cardiac function, Myocardial strain, Right ventricular volume

## Abstract

Functional analysis of the fetal cardiovascular system is crucial for the assessment of fetal condition. Evaluation of the right ventricle with standard 2D echocardiography is challenging due to its complex geometry and irregular muscle fibers arrangement. Software package TOMTEC 4D RV-Function is an analysis tool which allows assessment of right ventricular function based on volumetric measurements and myocardial deformation. The aim of this study was to determine the feasibility of this method in fetal echocardiography. The retrospective study was conducted in the high-flow Referral Center for Fetal Cardiology. We recorded 4D echocardiographic sequences of 46 fetuses with normal hearts. Following parameters were calculated: end-diastolic volume (EDV), end-systolic volume (ESV), stroke volume (SV) and ejection fraction (EF), right ventricle longitudinal free-wall (RVLS free-wall) and septal strain (RVLS septum). Tei index was calculated as a standard measure or RV function for comparison. 4D assessment was feasible in 38 out of 46 fetuses (83%). RV volumetric parameters—EDV, ESV and SV—increased exponentially with gestational age. Functional parameters—RV Tei index, EF and strains—were independent of gestational age. Mean EF was 45.2% (± 6%), RV free-wall strain was − 21.2% and RV septal strain was − 21.5%. There was a statistically significant correlation between septal and free-wall strains (r = 0.51, p = 0.001) as well as between EF and RV free-wall strain (r = − 0.41, p = 0.011). 4D RV assessment is feasible in most fetuses. Its clinical application should be further investigated in larger prospective studies.

## Background

Functional analysis of the fetal cardiovascular system is crucial for the evaluation of fetal condition. Congenital heart defects (CHD), arrhythmias, fetal hydrops, intrauterine growth restriction, twin to twin transfusion syndrome and many other conditions can lead to deterioration of fetal cardiac function. The assessment of the right ventricle—a dominant, systemic ventricle during prenatal life—is especially important, as its dysfunction is badly tolerated by the fetus.

Numerous parameters of fetal heart function have been described acquired from almost all currently available ultrasound modalities, both standard (2D imaging, Pulse Wave Doppler, Tissue Doppler) and newly developed (3D echocardiography, Speckle Tracking and Velocity Vector Imaging). Evaluation of right ventricular function with standard 2D echocardiography is challenging due to its complex geometry and irregular muscle fibers arrangement [1]. In fetuses, different loading conditions and “systemic” role of the right ventricle (RV) should be also considered, as well as small ventricular volumes and frequent changes of fetal position, further complicating the examination [2].

Software package TOMTEC 4D RV-Function is a new real-time three dimensional (RT3D) analysis tool which enables assessment of RV function based on volumetric measurements and myocardial deformation. This technique is based on the use of a combination of long- and short-axis views extracted from a right ventricle–focused 3D data set [3]. It allows to calculate end-diastolic volume (EDV), end-systolic volume (ESV), stroke volume (SV), ejection fraction (EF) and right ventricle longitudinal free-wall (RVLS free-wall) and septal strain (RVLS septum). 3D acquisition does not require geometric assumptions of RV and allows to measure RV volume directly, including inflow, outflow and apical regions [4]. Possibility of comparison between volumetric and functional parameters and higher accuracy of RV functional assessment in children were also observed [5]. Feasibility of this technique was proven in both pediatric and adult populations [6–9].

Studies in adult population comparing RT3DE with gold standard in RV assessment—cardiac magnetic resonance (CMR), conclude that RT3DE may become the method of choice for a simple and low-cost assessment of RV size and function [3, 10–12].

3D/4D ultrasound techniques have also been utilized in fetuses and shown to aid both screening and detailed echocardiographic assessment [13, 14]. 3D technique overcomes the limitation of geometric assumptions, especially in the RV volume assessment. The reliability and validity of this method for volume determination in both pre- and postnatal setting were proven in a series of publications by Herberg et al. [15–18]. Comparison between 4D STIC technique and 2D Doppler measurements of RV and LV volumes was presented in a paper by Rizzo et al. [19].

Hamill et al. tried to determine fetal cardiovascular parameters using spatiotemporal image correlation (STIC) and virtual organ computer-aided analysis (VOCAL) [20]. This method was used and presented previously by our team [21].

## Aim of the study

The aim of this study was to determine the feasibility of 4D echocardiography in the assessment of RV volume and function using the TOMTEC 4D RV-Function software in healthy fetuses. We also performed a review of the literature on that subject.

## Materials and methods

The retrospective study was performed from June to September 2016 in the high flow referral center for fetal cardiology.

Fetal echocardiographic examinations were performed with Philips Epiq7 (Philips, Bothell, Washington, USA) ultrasound machine according to the established rules using C9-2 convex transducer. 4D datasets were acquired in zoomed full-volume mode with the frame rate between 23 and 37 Hz (mean 28 Hz) using X5-1 xMATRIX array transducer and stored in DICOM format for offline analysis. Length of 3D-loops was 3 s.

Inclusions criteria were: normal fetal biometry and peripheral flows, normal fetal heart anatomy, normal fetal cardiovascular function, (here defined as CVPS = 10 and Tei index of RV and LV < 0.5 what is the reference value in our institution). 38 out of 46 recorded volumes of normal fetal hearts between 19 and 39 weeks of pregnancy (median 27) were suitable for further evaluation.

**Fig. 1:**
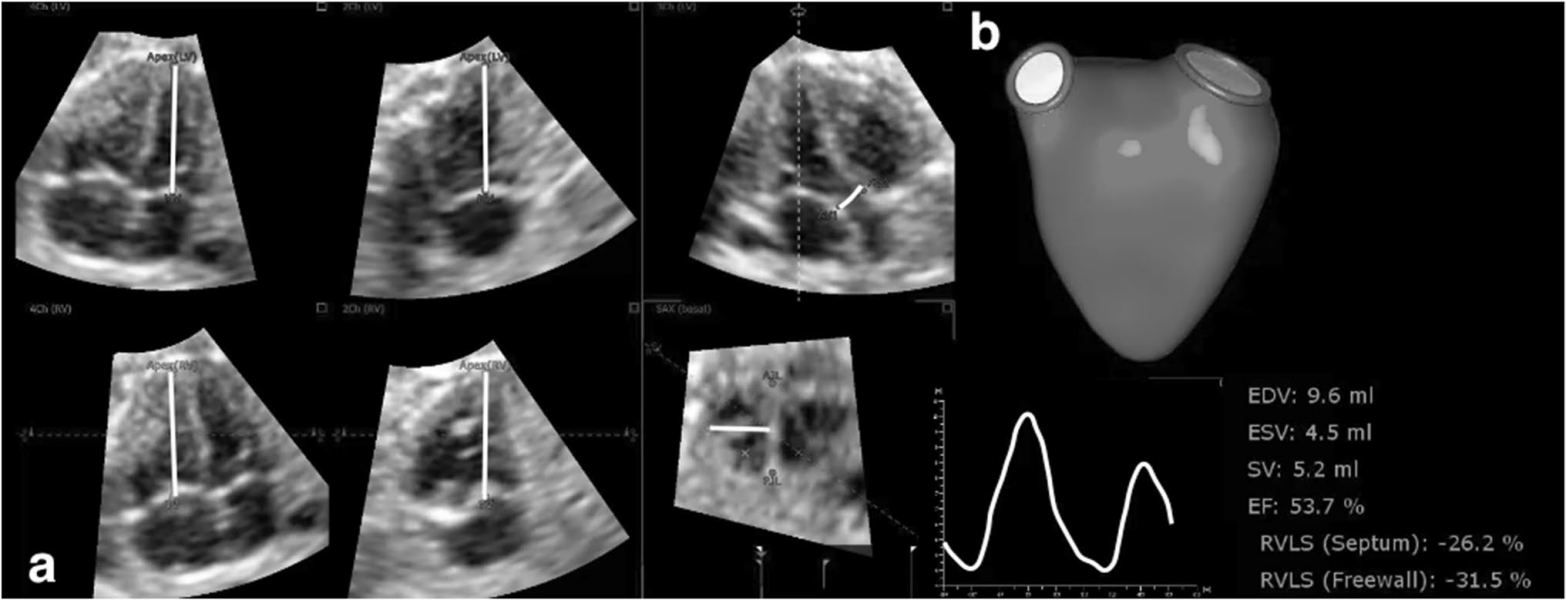
Assessment of RV volume and function using TOMTEC 4D RV-Function software: **a** location of landmarks in right and left ventricle. **b** example of right ventricle volumetric and functional parameters results

4D datasets were analyzed offline with TOMTEC 4D RV-Function 2.0 and Image Arena Version 4.6 (available in 2016) software. Volume images were oriented, adjusted, and displayed in sagittal, four-chamber, and coronal views. The end-diastolic (ED) frame was chosen as the largest chamber size and an end-systolic (ES) frame, as the smallest chamber size. The landmarks were placed in the center of tricuspid and mitral valves and at the RV and LV in two- and four-chamber views. In the three-chamber view aortic valve was identified. In short-axis view anterior and posterior junction points of the RV free wall with the interventricular septum and distance between RV free wall and interventricular septum were appointed (Fig. 1a). Endocardial contours were automatically traced and then manually adjusted. After manual adjustment of the endocardial borders the imaging software automatically detected the ventricular surfaces throughout the cardiac cycle. Ventricular volumes: right ventricle end-diastolic volume (EDV), end-systolic volume (ESV) were calculated and represented in a dynamic model. Stroke volume (SV) was calculated as SV = EDV − ESV. EF was calculated as EF = (EDV − ESV)/EDV. Right ventricle longitudinal free-wall (RVLS free-wall) and septal strain (RVLS septum) were also studied (Fig. 1b). We performed two sets of measurements by single investigator and an additional one by a second investigator to calculate intraobserver and interobserver variability. We used Bland Altman analysis.

Myocardial performance index (Tei index) measured by pulsed wave Doppler was used for comparison, as a standard reference method in the evaluation of fetal RV function (normal values below 0.5) [22]. Collected data were analyzed with StatSoft Statistica 13.1 software, using descriptive statistics, correlations, regression analysis and non-parametrical tests for comparisons due to the small size of the group. Pearson correlation coefficients were calculated to evaluate the relationship between gestational age and echocardiographic parameters.

## Results

4D RV assessment was feasible in 38 out of 46 fetuses (83%). Precise orientation of images and markers placement in the initial steps was crucial to achieve good alignment and tracing. Accurate detection of end-diastolic and end-systolic frame was also important.

Among the factors which hampered evaluation were: earlier gestational age (and therefore—smaller structures), poor image quality (difficult detection of endocardial contour) and low frame rate together with relatively high heart rate (less data for speckle tracking). Fetal movements impede data acquisition, but in all attempted cases it was possible to record stable 4D image containing complete heart cycle.

**Fig. 2:**
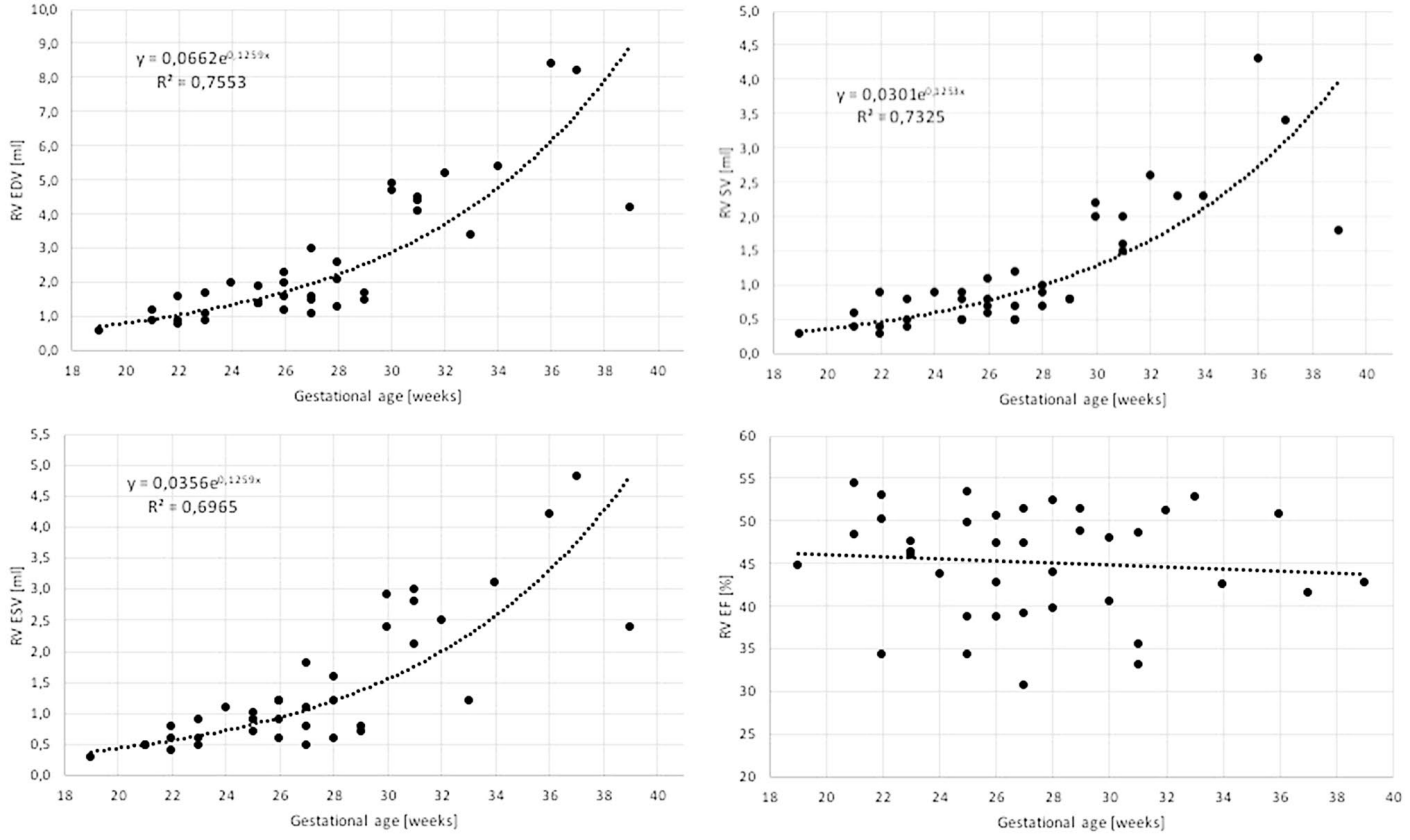
Correlation of right ventricle end-diastolic volume (EDV), end-systolic volume (ESV), stroke volume (SV) and ejection fraction (EF) with gestational age

**Fig. 3:**
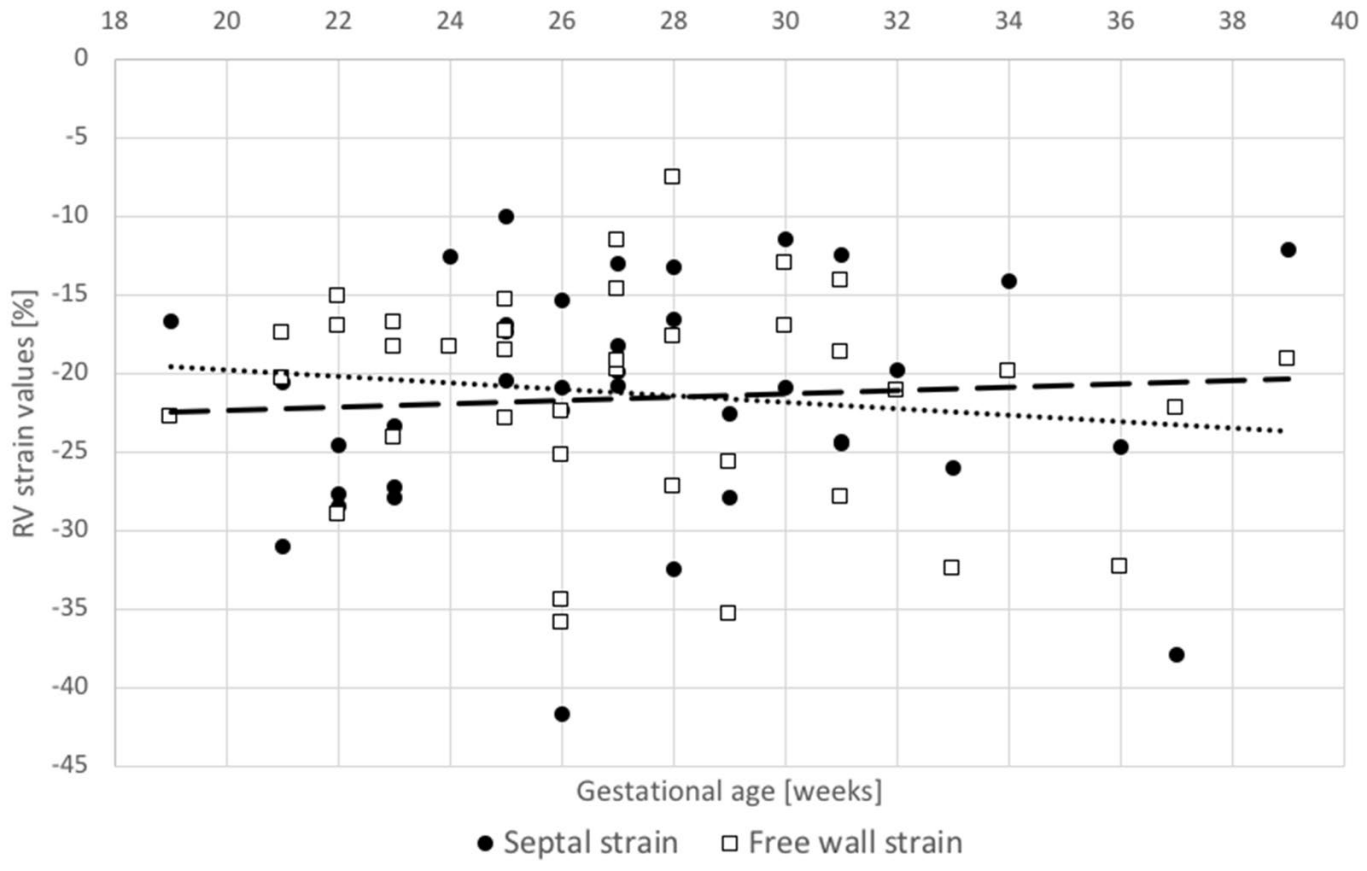
Correlation of RVLS free-wall and RVLS septum with gestational age

**Fig. 4:**
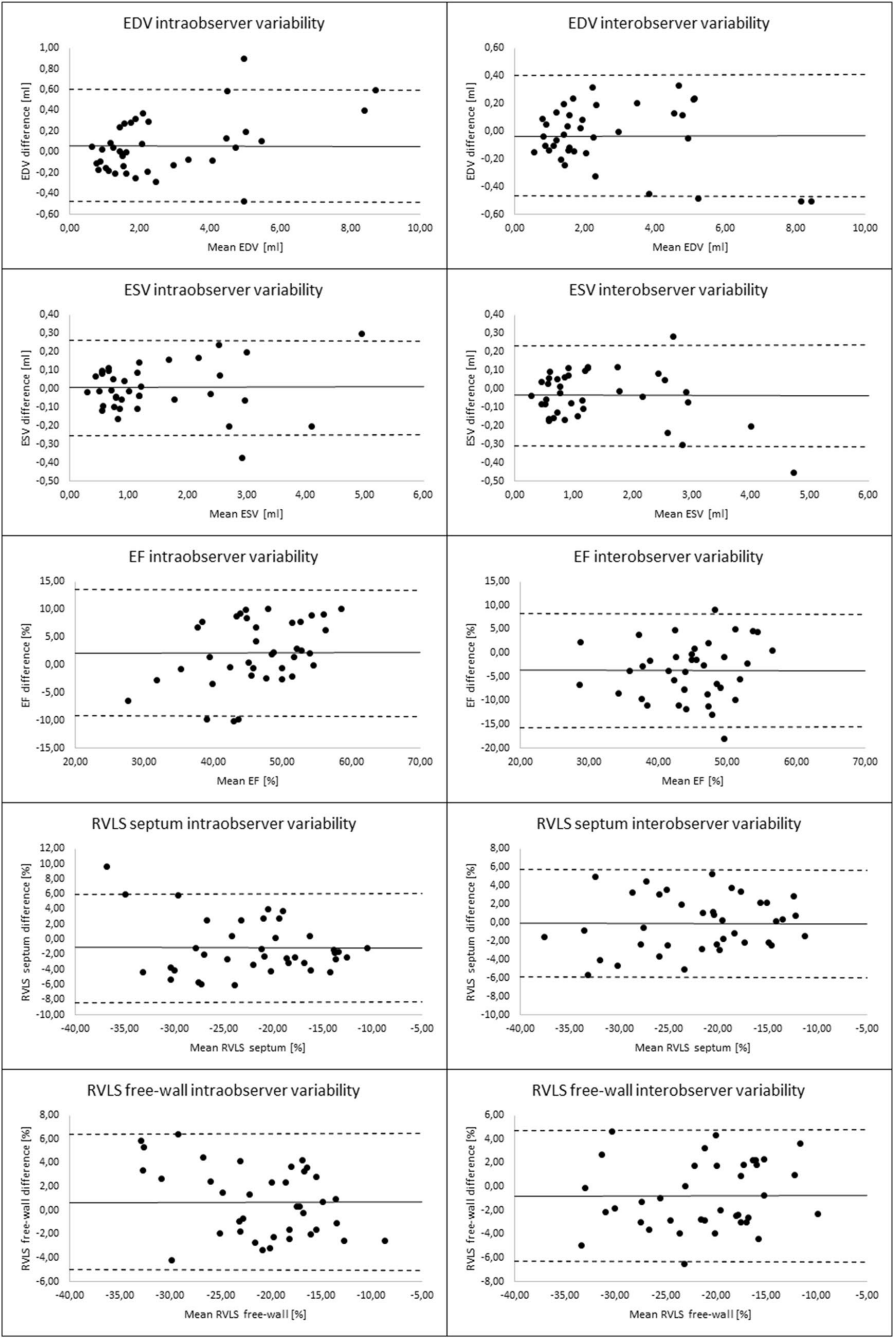
Bland–Altman plots for intra and interobserver agreement

In the group of 38 fetuses the volumetric parameters—RV EDV, ESV and SV—increased exponentially with gestational age (Fig. 2). Functional parameters—RV Tei index, EF and strains—were independent of gestational age (Figs. 2, 3). Mean EF was 45.2% (± 6.4%), RV free-wall strain was − 21.2% and RV septal strain was − 21.5%. With appropriate quality of datasets and meticulous alignment, the intra- and interobserver variability values were reasonable (Table 1 and Fig. 4). There was a statistically significant correlation between septal and free-wall strains (r = 0.51, p = 0.001), with a mean septal to free-wall ratio of 1.07 (0.58–2.22, SD 0.38). EF correlated significantly with RVLS free-wall strain (r = − 0.41, p = 0.011), the correlation with RV septal strain was weaker (r = − 0.23, p = 0.162) (Fig. 3). Tei index correlated poorly with both RV EF and strains.

**Table 1:** Parameters of cardiac function and intra- and interobserver variability

	Mean ± SD	Range	Intraobserver variability (bias ± SD)	Interobserver variability (bias ± SD)
WoG (weeks)	27 ± 5	19–39	–	–
EDV (ml)	–	0.6–8.4	0.06 ± 0.28	− 0.03 ± 0.22
ESV (ml)	–	0.3–4.8	0.01 ± 0.13	− 0.05 ± 0.14
EF (%)	45.2 ± 6.4	30.8–54.5	2.5 ± 5.8	3.6 ± 6.0
RVLS septum (%)	− 21.5 ± 7.3	− 10.0–(− 41.8)	− 1.0 ± 3.7	− 0.2 ± 3.0
RVLS free-wall (%)	− 21.2 ± 6.8	− 7.5–(− 35.9)	0.8 ± 2.9	− 0.7 ± 2.8

*WoG* weeks of gestation, *EF* ejection fraction, *RVLS septum* Right ventricle longitudinal septal strain, *RVLS free-wall* Right ventricle longitudinal free-wall, *EDV* end-diastolic volume, *ESV* end-systolic volume

## Discussion

4D echocardiographic imaging in the fetus is much more difficult than in the postnatal period, for several reasons. Assessed structures are smaller, not uniformly oriented and in a variable distance from the transducer. Moreover, there is no ECG tracing to aid the choice of end-systolic and end-diastolic frames—usually it is based on the M-mode imaging [23]. For the reasons mentioned above, the feasibility and quality of the analysis are dependent on the fetal age. Out of 5 fetuses aged 20 weeks or below, only in one case perfect visualization allowed for proper assessment. Our observation stays in contrast to Zheng et al. who suggested that the image quality did not change significantly after 16 weeks of gestation. We do agree that it is also related to fetal position, maternal acoustic window, and amniotic fluid quantity and clarity, in addition to gestational age [24]. Nevertheless, with the same image resolution and frame rate, bigger structure is easier to assess with speckle tracking technique—and so, larger hearts are visualized with more precision.

Volumetric parameters: In normal fetuses we observed an exponential increase of volumetric parameters: RV EDV, ESV and SV, which is consistent with most observations in the literature, however, some authors reported rather a linear trend of growth [14, 20, 24–26]. Data derived from different publications are difficult to compare, for methodological reasons—authors used different ultrasound machines, software and measurement techniques. The most utilized method was STIC 3D echocardiography followed by analysis with GE 4DView software and automatic (VOCAL, sonoAVC) or manual tracing [27]. Single authors used Philips RT3DE and QLAB or TOMTEC software (Table 2). TOMTEC software can also be used with different ultrasound machines, enabling offline analysis in such cases.

**Table 2:** Volumetric parameters of fetal RV-review of literature

Author	No of fetuses	GA	Technique	RV parameters	Conclusions
Simioni et al. [29]	265	20–34 + 6	STIC and VOCAL	EDV, ESV, SV	Exponential increase in SV
			Voluson 4DView	EF	Stable EF values (~ 63%)
Sun et al. [26]	123	22–35 + 6	iSTIC	EDV, ESV, SV	Linear increase in volumes
			Philips QLAB	EF	Stable EF values (~ 60%)
Hamill et al. [20]	184	19–40	STIC and VOCAL	EDV, ESV, SV	Exponential increase in volumes
			Voluson 4DView	EF	Linear decrease of EF values (~ 68–55%)
Zheng et al. [24]	52 normal and 9 CHD	17–34 + 6	RT3DE	EDV, ESV, SV	Exponential increase in volumes
			Tomtec	EF	Stable EF values (~ 73%)
Molina et al. [28]	140	12–34	STIC and VOCAL	SV, CO	Exponential increase in volumes
			Voluson 4DView		
Uittenbogaard et al. [25]	202	12–30	STIC and 3d Slice manual tracing method	EDV, ESV, SV, EF	Exponential quadratic increase in volumes
			Voluson 4DView		Stable EF values ~ 45%
Rizzo et al. [19]	40 normal and 16 FGR	20–22, 28–32, 26–34	STIC and VOCAL	SV	Linear increase in stroke volumes of both ventricles
			Voluson 4DView		
Rizzo et al. [27]	30 normal and 15 CHD	19–32	STIC and VOCAL or sonoAVC	ESV, EDV, SV	Increase in volumes with GA, regression data not given
			Voluson 4DView		Good correlation between volumes measured with VOCAL and sonoAVC (higher values with VOCAL)

*EDV* end-diastolic volume, *ESV* end-systolic volume, *SV* stroke volum

**Fig. 5:**
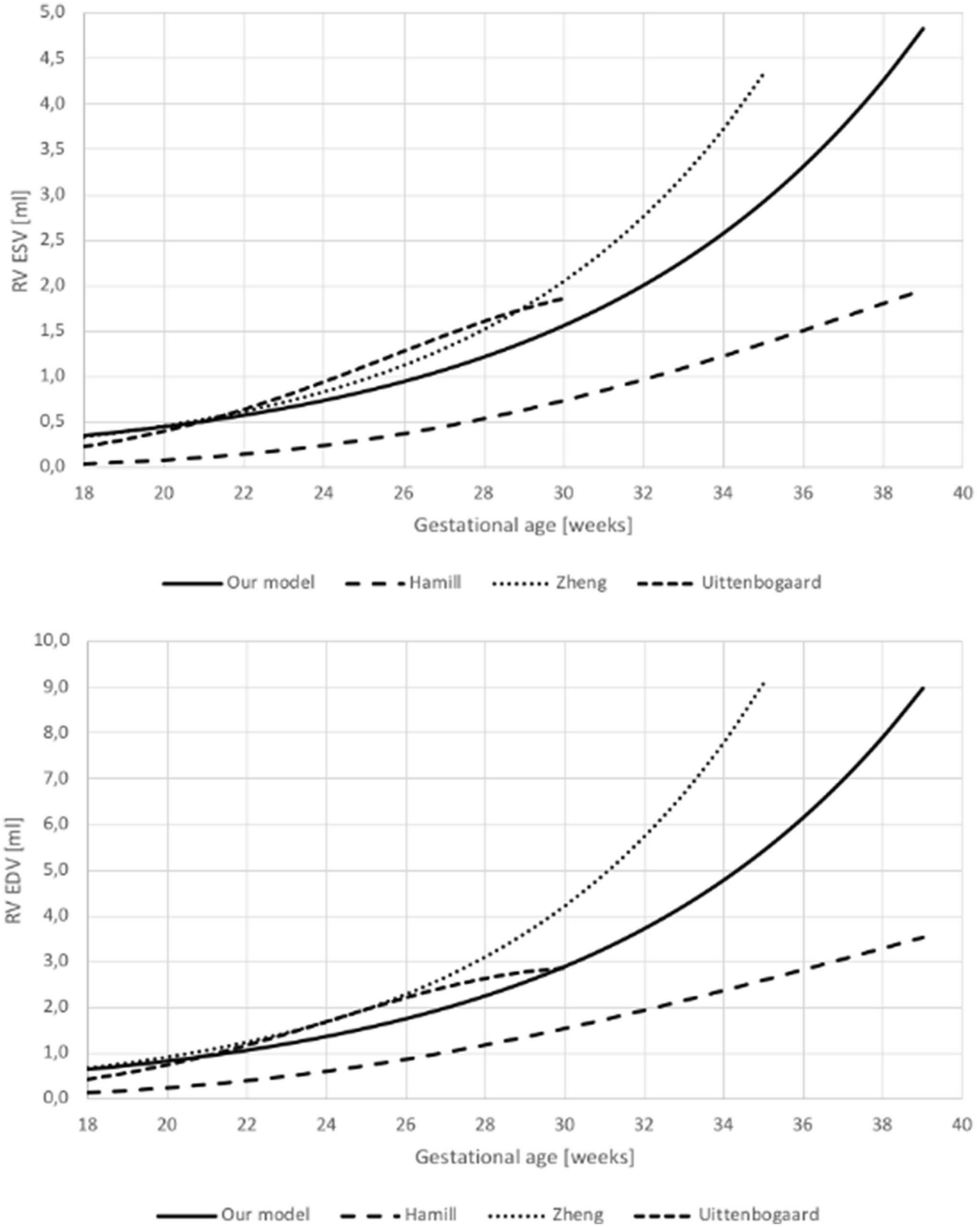
Comparison between our results of EDV and ESV measurement with data available in the literature

However, when comparing the data from available publications, there is a wide variance visible which cannot be attributed only to mentioned differences in methodology. Our data resemble most these of Molina et al. [28] and Simioni et al. [29], whereas values reported by Hamill [20] are lower, and by Zheng [24]—considerably higher (despite using the same method as in our study) (Fig. 5). This discrepancy may result from other factors, both patient’s and investigator’s dependent: inadequate visualization, low frame rate, small size of structures, vague endocardial tracing, inclusion (or not) of papillary muscles.

Ejection fraction, as a volume-derived parameter, suffers the same limitations as ventricular volumes. It is not surprising, then, that values reported by different authors are widely variable. RV EF values in our study were among the lowest reported, however, rather consistent with EF values measured postnatally. According to most of studies, including ours, EF values remain relatively stable throughout pregnancy, except for Hamill’s, who reported a decrease of EF with advancing gestational age. This again probably results from data postprocessing method and the precision of endocardial tracing.

Functional parameters: Strain parameters in the assessment of fetal RV function were measured mostly with 2D technique to date [30–33]. Kapusta et al. used offline 2D speckle-tracking analysis [31]. Di Salvo et al. and Ta-Shma et al. utilized automatic functional imaging (AFI) technique, which is a novel non-Doppler methodology based on 2-dimensional acoustic markers tracking, and measures myocardial deformation regardless of angle of interrogation [32, 33]. Achieved results were dependent on technique used and not consistent—authors observed stable value of strains [30], or their increase [32] or decrease [31] with gestational age (Table 3). In our study, RV free-wall strain and septal strain values were independent of GA. Due to different way of measurement, 2D strain values cannot be directly compared to these measured with 3D tools, however values reported by both techniques are similar (between − 20 and − 25%).

**Table 3:** Functional parameter of fetal RV-review of the literature

Author	No of fetuses	GA	Parameters	Conclusions
Erickson [30]	50	16–40	RV free wall strain RV septal strain	Decrease with GA − 24,8% (16–20 WoG) to − 19,4% (36–40 WoG) − 16,1% (16–20 WoG) to − 14,2% (36–40 WoG)
Kapusta [31]	49	20–24 and 30–34	Global RV strain	Decrease with GA − 25,35% in the 2nd trimester − 23,2% in the 3rd trimester
Di Salvo [32]	100	20–32	RV free wall strain RV septal strain	Increase with GA Mean 24% Mean 25%
Ta-Shma [33]	28	20–38	Global RV strain	21%, stable throughout pregnancy

*RV* right ventricle, *GA* gestational age, *WoG* weeks of gestation

To our knowledge, this is the first study that compares EF with strain value using a 3D technique in a fetus. As a result, we noticed that EF correlated significantly with RV free wall strain (r = − 0.41, p = 0.011), the correlation with RV septal strain was not significant (r = − 0.23, p = 0.162. This suggests that RV EF is dependent more on free wall translational movement and contractility, rather than septal contractility. We can also speculate that decrease in RV free-wall strain could be an early sign of abnormal RV function in the fetus, similar to what is shown in the postnatal population [34]. Tei index is a long-used measure to assess global (both systolic and diastolic) ventricle function. Its values remain relatively stable throughout pregnancy, therefore, in the group of normal fetuses, it is difficult to prove its correlation to RV EF or strain values. Further prospective studies including fetuses with ventricular dysfunction could show relative utility of these parameters in early detection and serial assessment.

Such relation was shown in study Solarz et al. where RV function was assessed in a group of children and adults after correction of Tetralogy of Fallot (TOF). They assessed RV function using a 2D technique and calculated strain, strain rate and ejection fraction in a group of operated patients and compared the results with a healthy cohort. They also observed that RVLS free-wall value correlated with a reduced EF (r = − 0.6) in a group of operated patients and was a proof of impairment of RV function [34].

The 3D technique in assessing EF is widely used in adult echocardiography, and the superiority of 3D over 2D was proven [7, 8]. Because of the systemic role of the RV in fetal life, its advanced assessment could be very important in different pathologies: from CHD, to pregnancy complications like IUGR [35], TTTS [36], intrauterine infection caused by preterm premature rupture of membranes [37] and maternal disease like diabetes mellitus [38, 39]. Wang et al. examined fetuses from pregnancies complicated by gestational diabetes mellitus (GDM) using 2D strain measurement. He observed that the peak systolic strain value of right ventricular free wall in the GDM group decreased significantly with a preserved value of EF [40]. Hamill et al. examined volumetric parameters of the ventricles in fetuses with umbilical artery pulsality index above the 95th percentile using STIC technique and compared with healthy fetuses. They observed that ventricular volumes are lower and the ejection fraction is higher as compared to normal fetuses [41]. However, there is still a need for large, prospective studies, which could overcome technical and methodological difficulties mentioned above and provide with unambiguous, good quality data on fetal ventricular function in both normal and pathological conditions.

The main limitations of our study are the small number of examined fetuses, as well as slow frame rate and image resolution relative to fetal heart rate and size. The main aim of our paper was to assess the feasibility of this new method. Study with large cohort of patients should be performed to determine reference values in fetal population.

## Conclusions

Assessment of fetal RV volume and function is feasible using TOMTEC 4D RV-Function software, especially in the 3rd trimester of pregnancy. The method has already proven its role in the postnatal population and seems promising in fetuses. However, data reported by different authors vary widely, as well as the machines and software used, therefore it is not yet possible to define universally accepted normal values of RV volumes and strains. This is a pilot study conducted on small number of fetuses within a wide range of gestational age, so there is a need for further studies to prove the utility of 4D echocardiography in the assessment of fetal ventricular function under pathological conditions.
